# Cooperative Fuzzy Games Approach to Setting Target Levels of ECs in Quality Function Deployment

**DOI:** 10.1155/2014/673563

**Published:** 2014-07-03

**Authors:** Zhihui Yang, Yizeng Chen, Yunqiang Yin

**Affiliations:** ^1^School of Management, Shanghai University, Shanghai 200444, China; ^2^College of Sciences, East China Institute of Technology, Nanchang, Jiangxi 330013, China

## Abstract

Quality function deployment (QFD) can provide a means of translating customer requirements (CRs) into engineering characteristics (ECs) for each stage of product development and production. The main objective of QFD-based product planning is to determine the target levels of ECs for a new product or service. QFD is a breakthrough tool which can effectively reduce the gap between CRs and a new product/service. Even though there are conflicts among some ECs, the objective of developing new product is to maximize the overall customer satisfaction. Therefore, there may be room for cooperation among ECs. A cooperative game framework combined with fuzzy set theory is developed to determine the target levels of the ECs in QFD. The key to develop the model is the formulation of the bargaining function. In the proposed methodology, the players are viewed as the membership functions of ECs to formulate the bargaining function. The solution for the proposed model is Pareto-optimal. An illustrated example is cited to demonstrate the application and performance of the proposed approach.

## 1. Introduction

Game theory is the discipline which studies multiple individuals implementing the corresponding strategy according to related strategies of other individuals under some situations. Sometimes we need to seek for the best strategies of each player taking into account that the others will also behave searching for their best. In this case, we call it as noncooperative games model. On the other hand, players just want to deal with the cooperation issues of the problem and consider how the agents allocate the benefits of their cooperation. This approach is called as the cooperative game [1, 2]. A cooperative game often assumes that each player is a part of a team and is willing to compromise his own payoff to improve the goal as a whole. A cooperative game proceeds with the intent that the team wants to allocate resources such that all players are as better off as possible, and an improvement in the payoff for one player does not result in a loss for other players. The bargaining scheme postulated by Nash yields a unique and optimal distribution of resources such that the arbitrated outcome is Pareto-optimal [1, 2].

Classical game theory is based on binary logic and the fully rational behavior assumption. Fuzzy logic is able to accommodate many of the binary-logic related dilemmas in classical game theory. In general, the players do not be having as fully rational decision makers in real games. Fuzzy logic is a tool for a formal representation of such behavior. Moreover, one of the outstanding limitations of the classical game theory is that it assumes that all the data are known exactly by all players. This assumption is often restrictive. In real world, it often happens that the players are not able to evaluate exactly the outcomes of different strategy profiles and their own preferences or the preferences of other players [3]. Therefore, to characterize the bounded rational behavior and games with imperfect or incomplete information, it is necessary to employ fuzzy logic into the game theory. Fuzzy logic was initiated by Zadeh for dealing with uncertainties [4]. From then on, fuzzy theory was extensively applied in many areas, such as decision sciences [5], control theory [6–8], and games theory [3, 9, 10].

Aubin [9] first studied the problem of fuzzy cooperative games. Dhingra and Rao [10] integrated the cooperative game theory and fuzzy set theory to yield a new optimization method. In this paper, the cooperative fuzzy game model, which was proposed by Dhingra and Rao [10], will be employed to QFD-based new product planning.

In the current economic globalization situation, more and more companies pay more attention to listen to the voice of customers. For many enterprises, the key to win competitive advantage is to develop the product with higher customer satisfaction, lower cost, and shorter product development cycle. The purpose of product innovation is that the designers can develop new products, which can attract customers and satisfy the demand of customers. Planning becomes essential in designing and manufacturing a new product efficiently at competitive cost within a short period of time [11]. As far product planning and development decisions are concerned extensively; the application of quality function deployment (QFD) has been applied in many areas. Originated in Japan in the late 1960s, QFD is a planning and problem-solving tool for translating customer requirements (CRs) into engineering characteristics (ECs) of a new product or service [12, 13]. QFD can help the designers systematically to determine ECs for developing a new product with maximum customer satisfaction. The QFD process includes four sets of matrices called houses of quality (HOQ) to relate CRs to product planning, parts deployment, process planning, and manufacturing operations [12, 13]. QFD is a breakthrough tool which can effectively reduce the gap between CRs and a new product or service.

The determination of the target levels of ECs is the core problem in QFD. The problem that setting target levels of ECs can be viewed as a game in which each player corresponds to the membership function of EC. Each player bargains with others to improve the payoff subjected to the limited resource. In the proposed methodology, the bargaining function is formulated as the geometric mean of the membership functions of ECs, and the development budget for the new product is fuzzified. Indeed, setting target levels of ECs is an optimization problem, in which the set of feasible solutions can be reduced to discrete, and the goal is to maximize the overall customer satisfaction. So setting target levels of ECs in QFD is also a combinatorial optimization problem.

The rest of this paper is organized as follows.  Section 2 reviews some related work about the determination of target levels of ECs in QFD.  Section 3 recalls the cooperative fuzzy game modeling approach proposed by Dhingra and Rao [10]. In Section 4, the fuzzy programming approach based on the fuzzy cooperative game model is put forward to determine the target levels of ECs. In Section 5, a motor car design is cited to illustrate the proposed methodology. Finally, the conclusions in this work are summarized in Section 6.

## 2. Related Work about QFD

In traditional QFD, the objective value of ECs is usually determined by the subjective experience of the QFD team. In order to determine the target levels of ECs objectively and accurately, the QFD team should develop the optimization model by taking the final importance of ECs and various constraints (cost, development time, technical feasibility, etc.) into account, where the goal is to help the QFD team to realize the overall customer satisfaction of new products catching up with or exceeding the competitors in the target market.

The determination of target levels of ECs under a fuzzy environment has gained extensive attention. Using a fuzzy ranking procedure, Zhou [14] investigated a mixed-integer linear programming model to optimize the target values of ECs. Fung et al. [15] developed a fuzzy inference model that features a fuzzy rule base to setting the target levels of ECs. Kim et al. [16] proposed a fuzzy multicriteria modeling approach to QFD planning in which fuzzy linear regression with symmetric triangular fuzzy numbers is used to estimate the functional relationships between CRs and ECs as well as among ECs. Taking into account the financial factors in the product design process, Tang et al. [17] developed a fuzzy formulation combined with a genetic-based interactive approach to QFD planning. To determine the target values of ECs, Bai and Kwong [18] proposed an inexact genetic algorithm approach to solve the model that takes the mutation along the weighted gradient direction as a genetic operator. Karsak [19] developed a fuzzy multiple objective programming approach that incorporates imprecise and subjective information inherent in the QFD planning process to determine the level of fulfillment of ECs.

There are two types of uncertainties in input in the QFD process: human perception and customer heterogeneity. To tackle the two types of uncertainties simultaneously, Chen et al. [20] developed two fuzzy expected value models to determine target values of ECs. By using dynamic programming proposed by Lai et al. [21], limited resources are allocated to the technical attributes. Y. Chen and L. Chen [22] developed a nonlinear-programming-based possibilistic regression approach. Fung et al. [23] developed a pair of hybrid linear programming models with asymmetric triangular fuzzy coefficients to estimate the functional relationships for product planning under uncertainties. Chen and Weng [24] proposed fuzzy goal programming models to determine the fulfillment levels of the ECs. Chen and Ngai [25] employed the method of imprecision (MoI) to perform multiple-attribute synthesis to generate a family of synthesis strategies by varying the value of *s*, which indicates the different compensation levels among ECs. A nonlinear-programming-based fuzzy regression approach was investigated in [26] to setting target levels of ECs. Chen and Ko [27] proposed fuzzy nonlinear-programming models based on Kano's concept to determine the fulfillment levels of parts characteristics with the aim of achieving the determined contribution levels of ECs for customer satisfaction. Delice and Güngör (2009) [28] investigated an approach to QFD processes to obtain the optimal solution from a limited number of design requirements alternatives with discrete value. Kwong et al. [29] investigated a generalized fuzzy least-squares regression approach to model customer satisfaction. Güngör et al. [30] used fuzzy analytic-network process (FANP) to determine the fulfillment levels of ECs. Liu [31] integrated fuzzy QFD and the prototype product selection model to develop a product design and selection approach that can substantially benefit developers in new product programming. Sener and Karsak [32] investigated an approach for determining target levels of ECs by integrating fuzzy linear regression and fuzzy multiple objective programming. Yang and Chen [33] employed fuzzy soft set theory to prioritize CRs and ECs in QFD. Jiang et al. [34] put forward a chaos-based fuzzy regression approach to model the relationships between customer satisfaction and ECs. Delice and Güngör (2013) developed a fuzzy mixed-integer goal programming model to setting the optimal discrete values of ECs [35]. Ko and Chen [36] established a new normalized relationship between CRs and ECs to improve the existing models' drawbacks and developed a fuzzy linear programming model to determine the optimal fulfillment levels of ECs. Considering several goals such as new product development time and cost, technological advancement, and manufacturability, Mungle et al. [37] proposed dynamical multiobjective evolutionary algorithm along with FANP and QFD to resolve product planning problem. Yuen [38] presented a hybrid framework of fuzzy cognitive network process, aggregative grading clustering, and QFD for the criteria evaluation and analysis in QFD.

The usefulness of these approaches is seriously limited because the performance of a complex product depends on some different, often conflicting, criteria that cannot be combined into a single measure of performance. Henceforth, a consideration of pursuing the maximization of the overall satisfaction of customers becomes a challenging problem to the design team. The process of setting the target levels of ECs is accomplished in a subjective adc manner or in a heuristic way. Due to many tradeoffs that may exist among implicit or plicit relationships between CRs and ECs and among ECs, these relationships cannot be identified using engineering knowledge. Due to cost and other resource constraints, tradeoffs are always needed. The purpose to setting target levels of ECs is to maximize the overall customer satisfaction. Therefore, there may be room for cooperation among ECs. In this study, the cooperative fuzzy game model, integrating the fuzzy set theory with the cooperative game theory, is employed to complex product planning.

## 3. Cooperative Games with Fuzzy Constraint

In this section, we recall the cooperative fuzzy game modeling approach proposed by Dhingra and Rao [10].

### 3.1. The Formulation of the Bargaining Function

Assume that there exists payoff functions $f_{i}(\vec{x})$, $\vec{x}\in S$ associated with each player *i*, where the set of alternatives *S* is convex and compact; the payoff of player *i* will be $f_{i}(\vec{x})$. These players bargain with each other and hope a trade such that the payoff functions are maximized. The bargaining function *B*(·) should satisfy the following inequality:

(1) min⁡(f1,f2,…,fm)<B(f1,f2,…,fm)<max⁡(f1,f2,…,fm),

where *B*(·) is a suitable operator that models a tradeoff among the goals $f_{i}=f_{i}(\vec{x})$, *i* = 1,2,…, *m*. In this study, the operator *B*(·) is set as the geometric mean with weight. Therefore, to determine a solution accepted by all players, the bargaining function $B(\vec{x})$ is formulated as follows:

(2) $$\begin{array}{c} B\left(\vec{x}\right)=\overset{m}{\underset{i=1}{\prod }}{\left(f_{i}(\vec{x})-f_{i}\left({\vec{x}}_{w}\right)\right)}^{1/m} \end{array}$$

for $\vec{x}\in S^{*}=\left\{X\in S|f_{i}(\vec{x})-f_{i}({\vec{x}}_{w})\geq 0\right\}\subset S$, where $f_{i}({\vec{x}}_{w})$ is the worst value of the payoff function $f_{i}(\vec{x})$ that player *i* is willing to accept.

The weights of all payoff functions in the bargaining function above are assumed to be equal. The generalized bargaining function is expressed as

(3) $$\begin{array}{c} B\left(\vec{x}\right)=\overset{m}{\underset{i=1}{\prod }}{\left(f_{i}\left(\vec{x}\right)-f_{i}\left({\vec{x}}_{w}\right)\right)}^{w_{i}}, \end{array}$$

where *w*_*i*_ denotes the weight of the payoff function $f_{i}(\vec{x})$, such that ∑_*i*=1_^*m*^ *w*_*i*_ = 1, 0 ≤ *w*_*i*_ ≤ 1, *i* = 1,2,…, *m*.

### 3.2. The Fuzzification of the Constraint

The constraint of an optimal problem often includes some crisp inequality and crisp equality. However, in some practical problem, these inequality and equality are often expressed vaguely. For example, the upper bound of the budget for a project is often expressed as “about one million dollars.” Thus the fuzzy logic is employed to characterize these inequality or equality. Assume that there are *n*_*fg*_ fuzzy inequalities and *n*_*fh*_ fuzzy equalities:

(4) $$\begin{array}{c} g_{i}\left(\vec{x}\right)\tilde{\leq }\;a_{i},\;i=1,2,\ldots ,n_{fg} \end{array}$$

(5) $$\begin{array}{c} h_{j}\left(\vec{x}\right)≅b_{j},\;j=1,2,\ldots ,n_{fh}. \end{array}$$

The fuzzy inequality (4) can be characterized by the membership function as follows:

(6) $$\begin{array}{c} \mu_{{\tilde{G}}_{i}}\left(\vec{x}\right)=\{\begin{array}{cc} 0, & g_{i}\left(\vec{x}\right)\geq a_{i}+\delta_{a_{i}},\\ \frac{a_{i}+\delta_{a_{i}}-g_{i}\left(\vec{x}\right)}{\delta_{a_{i}}}, & a_{i}<g_{i}\left(\vec{x}\right)<a_{i}+\delta_{a_{i}},\\ 1, & g_{i}\left(\vec{x}\right)\leq a_{i}, \end{array} \end{array}$$

where *δ*_*a*_*i*__ denotes the index that the upper bound of $g_{i}(\vec{x})$ can be improved.

The fuzzy equality (5) can be characterized by the membership function as follows:

(7) $$\begin{array}{c} \mu_{{\tilde{H}}_{j}}\left(\vec{x}\right)=\{\begin{array}{cc} 1-\left|\frac{h_{j}\left(\vec{x}\right)-b_{j}}{\tau_{b_{j}}}\right|, & b_{j}-\tau_{b_{j}}<h_{j}\left(\vec{x}\right)<b_{j}+\tau_{b_{j}},\\ 0, & \text{others}, \end{array} \end{array}$$

where *τ*_*b*_*j*__ denotes the index that the bound of $h_{j}(\vec{x})$ can be improved. The values of *δ*_*a*_*i*__ and *τ*_*b*_*j*__ can all be determined by the decision maker according to the experience or in a trial and error manner.

According to Bellman and Zadeh [5], let $\lambda =\min_{i,j}\left\{\mu_{{\tilde{G}}_{i}}(\vec{x}),\mu_{{\tilde{H}}_{j}}(\vec{x})\right\}$; then the model to determine the value of *λ* is formulated as follows:

(8a) $$\begin{array}{c} \max\;\lambda \end{array}$$

subject to

(8b) $$\begin{array}{c} \lambda \leq \mu_{{\tilde{G}}_{i}}\left(\vec{x}\right),\;i=1,2,\ldots ,n_{fg} \end{array}$$

(8c) $$\begin{array}{c} \lambda \leq \mu_{{\tilde{H}}_{j}}\left(\vec{x}\right),\;j=1,2,\ldots ,n_{fh}. \end{array}$$

### 3.3. The Formulation of the Cooperative Fuzzy Game Model

Combined the model (8a), (8b), and (8c) with the bargaining function expressed as (3), a cooperative fuzzy game model is formulated as follows:

(9a) $$\begin{array}{c} \max B\left(\vec{x}\right)+p\lambda \end{array}$$

subject to

(9b) $$\begin{array}{c} \lambda \leq \mu_{{\tilde{G}}_{i}}\left(\vec{x}\right),\;i=1,2,\ldots ,n_{fg} \end{array}$$

(9c) $$\begin{array}{c} \lambda \leq \mu_{{\tilde{H}}_{j}}\left(\vec{x}\right),\;j=1,2,\ldots ,n_{fh} \end{array}$$

(9d) $$\begin{array}{c} \vec{x}\in S^{*}=\left\{X\in S\;|\;f_{i}\left(\vec{x}\right)-f_{i}\left({\vec{x}}_{w}\right)\geq 0\right\}\subset S, \end{array}$$

where $B(\vec{x})=\prod_{i=1}^{m}{\left(f_{i}(\vec{x})-f_{i}({\vec{x}}_{w})\right)}^{w_{i}}$ and the parameter “*p*” in formula (9a) is determined by the decision maker.

As pointed out by Dhingra and Rao [10], the objective function $\max B(\vec{x})+p\lambda$ can reflect the tradeoff between the value of $B(\vec{x})$ and the degree of constraint violation 1 − *λ*.

### 3.4. Fuzzy Pareto-Optimality

The cooperative game is based on the concept of a Pareto-optimal solution. Considering a multiobjective problem as follows:

(10a) $$\begin{array}{c} \max\;f\left(\vec{x}\right)={\left(f_{1}\left(\vec{x}\right),f_{2}\left(\vec{x}\right),\ldots ,f_{m}\left(\vec{x}\right)\right)}^{T}, \end{array}$$

subject to

(10b) $$\begin{array}{c} \vec{x}\in S=\left\{\vec{x}\in R^{n}\;|\;g_{i}\left(\vec{x}\right)\leq a_{i},h_{j}\left(\vec{x}\right)=b_{j}\right\}, \end{array}$$

where $f_{1}(\vec{x}),f_{2}(\vec{x}),\ldots ,f_{m}(\vec{x})$ are objective functions, $\vec{x}$ is the vector of decision variables, and *S* is the set of feasible solutions.

For a multiple objective optimization problem with partly fuzzy constraints, the concept of Pareto-optimality used for optimization problems with crisp constraints needs to be revised to introduce the concept of a fuzzy Pareto-optimal solution. Thus Dhingra and Rao [10] extended the definition of Pareto-optimality as follows.

Let *f*_*i*_ : *R* ^*n*^ → *R*, *i* = 1,2,…, *m*, be the objective functions, $\mu_{{\tilde{G}}_{i}}:R^{n}\to [0,1]$, *i* = 1,2,…, *n*_*fg*_, and $\mu_{{\tilde{H}}_{j}}:R^{n}\to [0,1]$; *j* = 1,2,…, *n*_*fh*_ be the membership functions of fuzzy constraints. A solution ${\vec{x}}^{*}\in S$ is said to be fuzzy Pareto-optimal if and only if, for any ${\vec{x}}_{0}\in S$, $f_{i}({\vec{x}}_{0})\leq f_{i}({\vec{x}}^{*})$, *i* = 1,…, *m* with at least one stringent inequality, $\mu_{{\tilde{G}}_{i}}({\vec{x}}_{0})\geq \mu_{{\tilde{G}}_{i}}({\vec{x}}^{*})$, *i* = 1,2,…, *n*_*fg*_ with at least one stringent inequality, and $\mu_{{\tilde{H}}_{j}}({\vec{x}}_{0})\geq \mu_{{\tilde{H}}_{j}}({\vec{x}}^{*})$, *j* = 1,2,…, *n*_*fh*_ with at least one stringent inequality.

As pointed out by Dhingra and Rao [10], since the set of alternatives *S* is convex and compact, there exists an optimal solution of the problem (9a), (9b), (9c), and (9d) ${\vec{x}}^{*}\in S$ and it is fuzzy Pareto-optimal for the parameter *p* ≥ 0.

## 4. Programming Model Formulation

### 4.1. Notation

The notation used in this study can be summarized as follows:

 CR_*i*_ is the *i*th customer requirement, *i* = 1,2,…, *m*;
 EC_*j*_ is the *j*th engineering characteristic, *j* = 1,2,…, *n*;
 
*r*_*ij*_ is the strength of the correlation measure between CR_*i*_ and EC_*j*_;
 
*R* = (*r*_*ij*_) is the strength matrix between CRs and ECs;
 
*w*_*i*_ is the relative importance of CR_*i*_, *i* = 1,2,…, *m*;
 
**w** = (*w*_1_, *w*_2_,…, *w*_*n*_) is the vector of the relative importance of CRs;
 
*p*_*jk*_ is the strength of the correlation measure between EC_*j*_ and EC_*k*_;
 
**p**_*j*_ = (*p*_*j*1_, *p*_*j*2_,…, *p*_*jn*_) is the *j*th row vector of the matrix *P* = (*p*_*jk*_)_*n*×*n*_, *j* = 1,2,…, *n*;
 
*l*_*j*_ is the value of EC_*j*_, *j* = 1,2,…, *n*;
 
*x*_*j*_ is the level of attainment of EC_*j*_, 0 ≤ *x*_*j*_ ≤ 1, *j* = 1,2,…, *n*;
 
*v*_*j*_ is the relative importance of EC_*j*_, *j* = 1,2,…, *n*;
 
$C(\vec{x})$ is the total cost of product development, and it is a function varying with the vector $\vec{x}=(x_{1},x_{2},\ldots x_{n})$;
 
*C*_*F*_ is the fixed part of the development cost;
 
*C*_*v*_ is the variable part of the development cost;
 
*c*_*j*_ is the unit cost for *x*_*j*_, *j* = 1,2,…, *n*;
 
*T* is the budget of the product development; and
 
*t* is the index that the upper bound of *T* can be improved.

### 4.2. Normalization of the Values of ECs

To cover all types of inputs, *l*_*j*_ should be normalized to a scale [0,1]. The “smaller-the-better type (S-type)” and “larger-the-better type (L-type)” ECs can be normalized using the following formulas (11) and (12), respectively. Consider

(11) $$\begin{array}{c} x_{j}=\frac{l_{j}^{\max}-l_{j}}{l_{j}^{\max}-l_{j}^{\min}} \end{array}$$

(12) $$\begin{array}{c} x_{j}=\frac{l_{j}-l_{j}^{\min}}{l_{j}^{\max}-l_{j}^{\min}}. \end{array}$$

For L-type, *l*_*j*_^min⁡^ is the minimum value of EC_*j*_ that matches the performance of the main competitors and *l*_*j*_^max⁡^ is the maximized physical limit. Conversely, for S-type, *l*_*j*_^min⁡^ is the minimized physical limit minimum and *l*_*j*_^max⁡^ is the maximum value of EC_*j*_ that matches the performance of the main competitors.

### 4.3. Calculation of *v*_*j*_

The relative importance of ECs, *v*_*j*_, *j* = 1,2,…, *n*, can be calculated as

(13a) $$\begin{array}{c} v_{j}=\frac{v_{j}^{\prime }}{\sum_{j=1}^{n}v_{j}^{\prime }}, \end{array}$$

(13b) $$\begin{array}{c} v_{j}^{\prime }=wRp_{j}^{T}. \end{array}$$

### 4.4. The Development Cost with Its Fuzzification

The development cost $C(\vec{x})$ can be viewed as the sum of the fixed cost *C*_*F*_ and the variable cost *C*_*v*_, where *C*_*v*_ is the sum of *x*_*j*_ with the unit cost *c*_*j*_. Therefore the calculation formula of the development cost $C(\vec{x})$ can be expressed as follows:

(14) $$\begin{array}{c} C\left(\vec{x}\right)=C_{F}+C_{V}=C_{F}+\overset{n}{\underset{j=1}{\sum }}c_{j}x_{j}. \end{array}$$

If the total cost of product development $C(\vec{x})$ is constrained to a budget *T*, it can be expressed as $C(\vec{x})\leq T$.

In practical problem, the design team often needs to improve the upper limit of the budget to enhance the levels of ECs. Considering the budget *T* that can be expanded to *T* + *t* (*t* > 0) as it is needed, where *t* denotes the distance by which the upper bound of the budget can be moved, we can fuzzify the cost constraint as

(15) $$\begin{array}{c} \mu_{\tilde{C}}\left(\vec{x}\right)=\{\begin{array}{cc} 1, & C\left(\vec{x}\right)<T,\\ \frac{T+t-C\left(\vec{x}\right)}{t}, & T\leq C\left(\vec{x}\right)\leq T+t,\\ 0, & C\left(\vec{x}\right)>T+t. \end{array} \end{array}$$

### 4.5. Development of the Programming Model

In this subsection, we will develop a model to determine the target values of ECs, in which the objective of the programming model is to maximize the overall customer satisfaction and to exceed the main competitors.

The overall customer satisfaction can be obtained by aggregating the membership functions of the *x*_*j*_, *u*_*j*_(*x*_*j*_), *j* = 1,2,…, *n*, and their relative weights *v*_*j*_, *j* = 1,2,…, *n*. Existing research often utilizes the sum with weight to aggregate *μ*_*j*_(*x*_*j*_) and *v*_*j*_. As introduced in Section 3, the bargaining function $B(\vec{x})=\prod_{i=1}^{m}{\left(f_{i}(\vec{x})-f_{i}({\vec{x}}_{w})\right)}^{w_{i}}$ is similar to the geometric mean with weight. So we formulate the bargaining function $B(\vec{x})$ as

(16) $$\begin{array}{c} B\left(\vec{x}\right)=\overset{n}{\underset{j=1}{\prod }}u_{j}{\left(x_{j}\right)}^{v_{j}}, \end{array}$$

where the payoff function of the player *j* is *u*_*j*_(*x*_*j*_) and its worst value is zero. Indeed $B(\vec{x})=\prod_{j=1}^{n}u_{j}{\left(x_{j}\right)}^{v_{j}}$ is the geometric mean with weight for the membership function *u*_*j*_(*x*_*j*_), *j* = 1,2,…, *n*, and it also can represent the overall customer satisfaction. This function can realize the tradeoff amongst ECs. Therefore, the programming model is formulated as follows:

(17a) $$\begin{array}{c} \max\;B\left(\vec{x}\right)+p\lambda \end{array}$$

subject to

(17b) $$\begin{array}{c} \lambda \leq \mu_{\tilde{C}}\left(\vec{x}\right) \end{array}$$

(17c) $$\begin{array}{c} \vec{x}=\left(x_{1},x_{2},\ldots ,x_{n}\right)\in {\left[0,1\right]}^{n}, \end{array}$$

where $B(\vec{x})=\prod_{j=1}^{n}u_{j}{\left(x_{j}\right)}^{v_{j}}$ and the parameter “*p*” is determined by the decision maker.

Since the feasible set [0,1]^*n*^ is convex and compact, there exists a fuzzy Pareto-optimal solution of the problems (17a), (17b), and (17c) ${\vec{x}}^{*}\in S$ for the parameter *p* ≥ 0.

## 5. An Illustrated Example

### 5.1. Building a HOQ for the Motor Car

In QFD, target values of ECs identify the definitive and quantitative technical specifications to satisfy CRs. The main objective of QFD-based product planning is to determine the target values of ECs for a new product to maximize the overall customer satisfaction with the given limited resources. In this section we will illustrate the proposed methodology by using a design of motor car (Chen et al. 2005, 2008) [20, 25].

A corporation is developing a new type of motor car. As depicted in Table 1, five CRs are identified to represent the biggest concerns of the customers. They are “reducing the noise of the car” (CR_1_), “enhancing the acceleration” (CR_2_), “saving fuel” (CR_3_), “improving security” (CR_4_), and “seat comfort” (CR_5_). Their relative weights are determined by analytic hierarchy process (AHP) and listed in the second column of the Table 1. Once CRs are identified, the ECs are tabulated in the house of quality in order to satisfy CRs. Based on the design team's experience and expert knowledge on this product, five ECs are determined, that is, “reducing the noise of the exhaust system” (EC_1_), “increasing the horsepower of the engine” (EC_2_), “reducing the amount of fuel per mile” (EC_3_), “increasing the controlling force of the braking system” (EC_4_), and “enlarging the space of the seat” (EC_5_). These ECs are measured in units of dB, Horsepower, Gallon, Kg, and M^3^, respectively. The negative and positive sign on ECs mean that the design team hopes to reduce and increase the target values of ECs, that is, EC_1_, EC_3_ belong to “S-type,” and others belong to “L-type”. The QFD team will identify the strength of the relationship between CRs and ECs. These relationships are indicated in the relationship matrix between the CRs and ECs. According to formulas (13a) and (13b), the relative importance of the five ECs is calculated as (*v*_1_, *v*_2_, *v*_3_, *v*_4_, *v*_5_) = (0.30,0.19,0.24,0.19,0.08), which are shown in the bottom of the HOQ. The level values of ECs of five main competitors, Comp1, Comp2, Comp3, Comp4, and Comp5, are shown in the HOQ. The objective of the design team is to determine the target values of ECs for our product, so that the overall customer satisfaction of our product can exceed the main competitors.

The HOQ for the motor car design is shown in Table 1.

### 5.2. Normalizing the Values of ECs

The values of EC_1_ and EC_3_ for the five competitors are normalized by using (11), and the values of EC_2_, EC_4_, and EC_5_ for the five competitors are normalized are by using (12). The normalization results for the five ECs of the five competitors are listed in Table 2.

### 5.3. Representing Design Uncertainty and Fuzzy Cost

To represent the design uncertainty, Chen and Ngai [25] defined a kind of membership function for a trapezoidal fuzzy number. The membership functions of the five ECs formulated by Chen and Ngai [25] are as

(18) $$\begin{array}{c} u_{1}\left(x_{1}\right)=x_{1}^{0.2},\;0\leq x_{1}\leq 1\\ u_{2}\left(x_{2}\right)=x_{2}^{2},\;0\leq x_{2}\leq 1\\ u_{3}\left(x_{3}\right)=x_{3}^{0.2},\;0\leq x_{3}\leq 1\\ u_{4}\left(x_{4}\right)=x_{4},\;0\leq x_{4}\leq 1\\ u_{5}\left(x_{5}\right)=x_{5}^{4},\;0\leq x_{5}\leq 1. \end{array}$$

**Figure 1:**
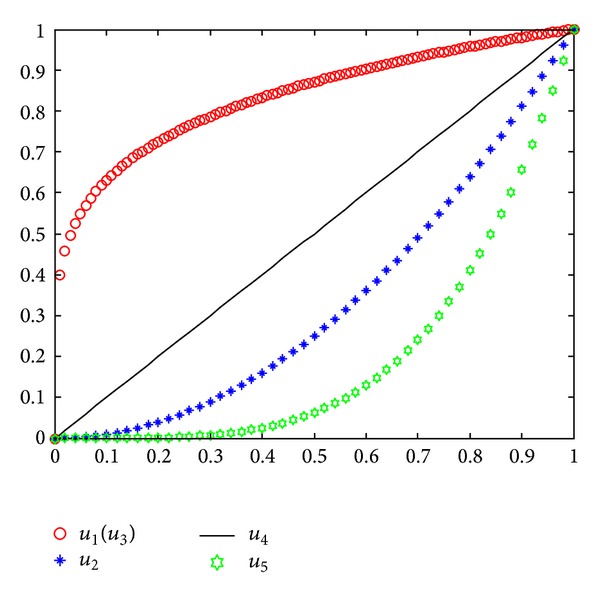
Membership functions of ECs.

The above membership functions of the five ECs are depicted in Figure 1.

The fixed cost *C*_*F*_ for the basic design, the unit cost for the five ECs, the development budget *T*, and its telescopic indicator *t* are listed in Table 3.

Therefore, the development cost $C(\vec{x})$ for the motor car design can be expressed as

(19) $$\begin{array}{c} C\left(\vec{x}\right)=50+25x_{1}+10x_{2}+15x_{3}+10x_{4}+8x_{5}. \end{array}$$

Considering the upper bound of the budget to be improved as it is needed, the membership function of the fuzzy cost can be formulated as

(20) $$\begin{array}{c} \mu_{\tilde{C}}\left(\vec{x}\right)=\{\begin{array}{cc} 1, & C\left(\vec{x}\right)<75,\\ \frac{80-C\left(\vec{x}\right)}{80-75}, & 75\leq C\left(\vec{x}\right)\leq 80,\\ 0, & C\left(\vec{x}\right)>80. \end{array} \end{array}$$

### 5.4. Results and Discussion

#### 5.4.1. Analysis of Results

According to the formulas (16) and (18), the results about the membership degree for ECs and the overall customer satisfaction of the five competitors are listed in Table 4, where the overall customer satisfaction of Comp_3_ is 0.7274, which is largest amongst five competitors.

**Figure 2:**
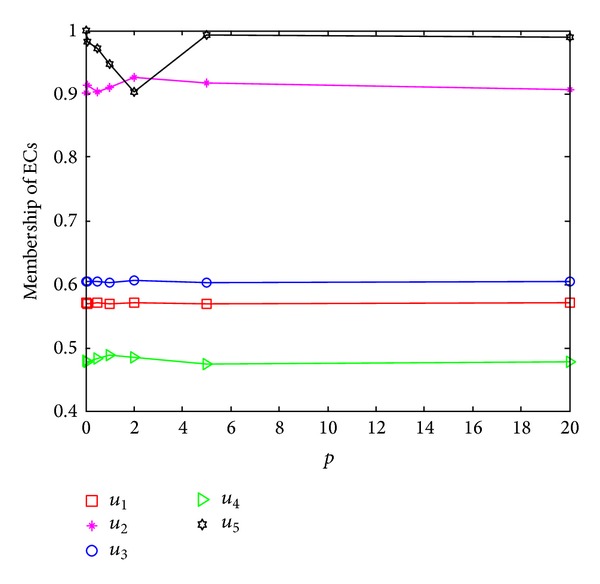
Membership of the five ECs with different value of “*p*.”

Combined the formulas (16), (18), and (20), the solution for the cooperative fuzzy game models (17a), (17b), and (17c) with different value of the parameter “*p*” is tabulated as Table 5. From Table 5 and Figure 2, it can be seen that the total cost is still 75, but the varying of the parameter “*p*” can facilitate the good performance in one EC to compensate for poor performance in other ECs slightly.

The membership degree for ECs and their overall customer satisfaction with different value of the parameter “*p*” are shown in Table 6.

From Table 6 it can be seen that the overall customer satisfaction obtained from the proposed method is always 0.6383 though the value of the parameter “*p*” varies from 0 to 20. Indeed, as introduced in Section 3, because the feasible set [0,1]^*n*^ is convex and compact, there exists a fuzzy Pareto-optimal solution of the problems (17a), (17b), and (17c) ${\vec{x}}^{*}\in S$ for the parameter *p* ≥ 0.

Moreover, the result in Table 6 shows that the overall customer satisfaction ($B(\vec{x})=0.6383$) obtained from the proposed method exceeds four competitors only smaller than Comp_3_ (0.7274).

#### 5.4.2. Further Discussion

As discussed in Section 5.4.1, when the budget is limited as 75, the overall customer satisfaction $B(\vec{x})$ is 0.6383, which is Pareto-optima. If we hope that the overall customer satisfaction of our new product exceeds all competitors, we must improve the budget. So we set the budget *T* as 70 and 80, respectively, and the telescopic indicator *t* is still set as 5. For comparison, the results with different budget are listed in Table 7 when the parameter *p* = 1.

From Table 7, it can be seen that the overall customer satisfaction of our new product can exceed all competitors when we set the budget as 80.

## 6. Conclusion

In this study, to enhance the overall customer satisfaction, a cooperative game fuzzy framework is developed to determine the target values of the ECs in QFD, where each player corresponds to the membership function of ECs. The formulation of the bargaining function is the key in the proposed approach. A motor car product design is cited to illustrate the proposed approach. Results show that the overall customer satisfaction for the ECs obtained from the proposed methodology can exceed the main competitors. The advantage of the proposed methodology is that the solution for the model with limited resources is Pareto-optimal. Meanwhile, the varying of the parameter “*p*” can facilitate the good performance in one EC to compensate for poor performance in other ECs. It is important to note that there is no model that employs the cooperative fuzzy game modeling approach over QFD analysis.

Existing methods for determining the target levels of ECs in QFD often consider CRs and the relationships between CRs and ECs acquired previously. Therefore, it is very difficult that a new product or service fully meets customer expectations when it is ready to market. In order to tackle this problem, it is necessary to embed the dynamics customer requirements into QFD. For future research, we would like to develop fuzzy game framework to determine the target levels of ECs of the new product by considering future requirements that meet customer needs.

## Figures and Tables

**Table 1:** The house for the motor car, Chen and Ngai [25].

	Correlation matrix					
	ECs	− EC_1_ *x*_1_	+ EC_2_ *x*_2_	− EC_3_ *x*_3_	+ EC_4_ *x*_4_	+ EC_5_ *x*_5_
	EC_1_	1	0	0	0	0
	EC_2_	0	1	0.2	0	0
	EC_3_	0	0.2	1	0	0
	EC_4_	0	0	0	1	0
	EC_5_	0	0	0	0	1

CRs	Weights of CRs	Relationship matrix				

CR_1_	0.31	1	0	0.2	0	0
CR_2_	0.25	0	0.6	0	0	0
CR_3_	0.16	0	0	1	0	0
CR_4_	0.20	0	0	0	1	0
CR_5_	0.08	0	0	0	0	1

	Technical matrix					
	Units	dB	Horsepower	Gallon	Kg	M^3^

	Comp_1_	80	75	0.042	25	0.18
	Comp_2_	65	65	0.034	24	0.20
	Comp_3_	65	80	0.028	23	0.18
	Comp_4_	75	60	0.032	15	0.14
	Comp_5_	95	80	0.030	20	0.19
	min	60	55	0.027	13	0.12
	max	100	90	0.044	27	0.21

	Relative weights of ECs	0.30	0.19	0.24	0.19	0.08

**Table 2:** Normalization of the ECs of the five competitors.

	*x*_1_	*x*_2_	*x*_3_	*x*_4_	*x*_5_
Comp_1_	0.5000	0.5714	0.1176	0.8571	0.6667
Comp_2_	0.8750	0.4286	0.5882	0.7857	0.8889
Comp_3_	0.8750	0.7143	0.9412	0.7143	0.6667
Comp_4_	0.6250	0.1429	0.7059	0.1429	0.2222
Comp_5_	0.1250	0.7143	0.8235	0.5000	0.7778

**Table 3:** The fixed cost, unit cost, and budget (units).

*C*_*F*_	*c*_1_	*c*_2_	*c*_3_	*c*_4_	*c*_5_	*T*	*t*
50	25	10	15	10	8	75	5

**Table 4:** Membership degree for ECs and overall customer satisfaction of the five competitors.

	μ_1_(*x*_1_)	μ_2_(*x*_2_)	μ_3_(*x*_3_)	μ_4_(*x*_4_)	μ_5_(*x*_5_)	$B(\vec{x})$
Comp_1_	0.8706	0.3265	0.6518	0.8571	0.1976	0.5969
Comp_2_	0.7936	0.1837	0.8993	0.7857	0.6243	0.6064
Comp_3_	0.9736	0.5495	0.9880	0.7143	0.1976	0.7274
Comp_4_	0.9103	0.0204	0.9327	0.1429	0.0024	0.1946
Comp_5_	0.6598	0.5102	0.9619	0.5000	0.3660	0.6225

**Table 5:** Target values of ECs with different value of the parameter “*p*.”

*p*	*x*_1_	*x*_2_	*x*_3_	*x*_4_	*x*_5_	$C(\vec{x})$
0	0.0603	0.9490	0.0806	0.4794	1.0000	75
0.05	0.0599	0.9557	0.0804	0.4775	0.9954	75
0.50	0.0609	0.9497	0.0805	0.4832	0.9925	75
1	0.0602	0.9533	0.0796	0.4878	0.9862	75
2	0.0606	0.9621	0.0813	0.4844	0.9749	75
5	0.0600	0.9579	0.0799	0.4738	0.9981	75
20	0.0605	0.9522	0.0805	0.4782	0.9971	75

**Table 6:** The overall customer satisfaction with different value of the parameter “*p*.”

*p*	*u*_1_(*x*_1_)	*u*_2_(*x*_2_)	*u*_3_(*x*_3_)	*u*_4_(*x*_4_)	*u*_5_(*x*_5_)	$B(\vec{x})$
0	0.5702	0.9006	0.6043	0.4794	1.0000	0.6383
0.05	0.5696	0.9133	0.6041	0.4775	0.9817	0.6383
0.50	0.5715	0.9019	0.6041	0.4832	0.9705	0.6383
1	0.5701	0.9088	0.6028	0.4878	0.9458	0.6383
2	0.5709	0.9256	0.6054	0.4844	0.9033	0.6383
5	0.5696	0.9175	0.6033	0.4738	0.9924	0.6383
20	0.5706	0.9067	0.6042	0.4782	0.9883	0.6383

**Table 7:** Results with different budget when *p* = 1.

*T*	*x*_1_	*x*_2_	*x*_3_	*x*_4_	*x*_5_	$B(\vec{x})$
70	0.0480	0.7607	0.0638	0.3824	0.8015	0.5109
75	0.0602	0.9533	0.0796	0.4878	0.9862	0.6383
80	0.0967	1.0000	0.1291	0.7646	1.0000	0.7487
